# ModFOLD6: an accurate web server for the global and local quality estimation of 3D protein models

**DOI:** 10.1093/nar/gkx332

**Published:** 2017-04-29

**Authors:** Ali H. A. Maghrabi, Liam J. McGuffin

**Affiliations:** School of Biological Sciences, University of Reading, Whiteknights, Reading RG6 6AS, UK

## Abstract

Methods that reliably estimate the likely similarity between the predicted and native structures of proteins have become essential for driving the acceptance and adoption of three-dimensional protein models by life scientists. ModFOLD6 is the latest version of our leading resource for Estimates of Model Accuracy (EMA), which uses a pioneering hybrid quasi-single model approach. The ModFOLD6 server integrates scores from three pure-single model methods and three quasi-single model methods using a neural network to estimate local quality scores. Additionally, the server provides three options for producing global score estimates, depending on the requirements of the user: (i) ModFOLD6_rank, which is optimized for ranking/selection, (ii) ModFOLD6_cor, which is optimized for correlations of predicted and observed scores and (iii) ModFOLD6 global for balanced performance. The ModFOLD6 methods rank among the top few for EMA, according to independent blind testing by the CASP12 assessors. The ModFOLD6 server is also continuously automatically evaluated as part of the CAMEO project, where significant performance gains have been observed compared to our previous server and other publicly available servers. The ModFOLD6 server is freely available at: http://www.reading.ac.uk/bioinf/ModFOLD/.

## INTRODUCTION

Predicted three-dimensional (3D) models of proteins are now routinely relied upon to drive research across the life sciences, mainly due to the expense and time limitations of determining structures experimentally. 3D models are comparatively quick to produce and can often be of sufficiently high quality. However, with all predictions there is some level of uncertainty, and therefore accurate methods for model quality assessment have become necessary for driving the acceptance of structure prediction methods. Essentially, relying on a 3D model of a protein without an estimate of its accuracy is tantamount to relying on a sequence alignment without an E-value. Thus, the development of 3D model Quality Assessment (QA) tools has become an important area of research in itself. Numerous methods have been developed over the years in an attempt to provide users with scores that will give them confidence in their 3D models and allow them identify any potentially suspect regions.

The model quality assessment field has its roots in early structure validation tools (1–3). Such tools can be used to perform basic stereochemical checks, and they are very useful in identifying unusual geometric features in a model. However, such methods are not able to produce a single global score that can be used for ranking alternative models or discriminating good models from bad (often bad models will still have good stereochemistry). Modern methods for QA can be classified into three broad categories: pure-single model methods, which consider only information within an individual model (4–11), clustering/consensus approaches (12–16), which can only be used if you have multiple alternative models built for the same protein target, and quasi-single model methods (17,18), which can score an individual model against a pool of alternative models generated from the target sequence. Each approach has its advantages and disadvantages. Clustering methods have been far more accurate than pure single-model methods, but are more computationally intensive and do not work when very few similar models are available, which is often the case in real life research scenarios. Pure-single model methods are less accurate overall, but they are more rapid, they produce consistent scores for single or few models at a time and they often perform better at model ranking and selection.

Quasi-single model methods attempt to provide comparable accuracy to clustering methods, while addressing real-life needs of researchers with few/single models. We initially implemented a quasi-single model approach with our ModFOLD3 method (18), which generated reference sets of models from the target sequence, using IntFOLD-TS (19), for comparison with the submitted model using ModFOLDclust2 (16). The method has since undergone a number of updates: ModFOLD4 (17), which makes use of IntFOLD2-TS (20) models, and ModFOLD5, which makes use of IntFOLD3-TS (21) models. Each of these quasi-single model versions of ModFOLD have been ranked among the top performing methods in the quality assessment categories of the recent CASP experiments (22,23) and have undergone incremental improvements in accuracy. By some measures, the quasi-single model methods have been competitive with the predictive power offered by clustering-based methods, as well as being capable of making predictions for a single model at a time. While the ModFOLD server has been a pioneer of the quasi-single model approach and a leader in terms of prediction performance, it has fallen short in some aspects, such as model selection. Furthermore, there is still significant room for improvement in many aspects of quality assessment.

Here we describe significant major updates to the ModFOLD server. The server has been popular with modellers around the world, having completed ∼200 000 quality assessment jobs for ∼9000 unique users. The latest version, ModFOLD6, operates solely in single model mode, deploying a novel hybrid pure/quasi-single model QA algorithm. In addition to interface updates, in this paper we will also briefly describe the major modifications to the prediction algorithm, which have led to significant performance gains in both local and global model quality predictions, allowing us to maintain our position as a leading prediction group. The main changes under the hood have been the addition of several new local scoring inputs, a new neural network (NN) architecture and alternative optimized global scores for different use cases. On the front end submission page, users are now given three alternative choices for optimized global model quality scoring, depending on whether their preference is for optimal model selection (the best models are ranked at the very top), predicting absolute values (the predicted scores closely reflect the observed scores) or more balanced performance for the two use cases. We also report on the independent benchmarking of the server for the recent CASP12 experiment and ongoing CAMEO project.

## MATERIALS AND METHODS

**Figure 1.**
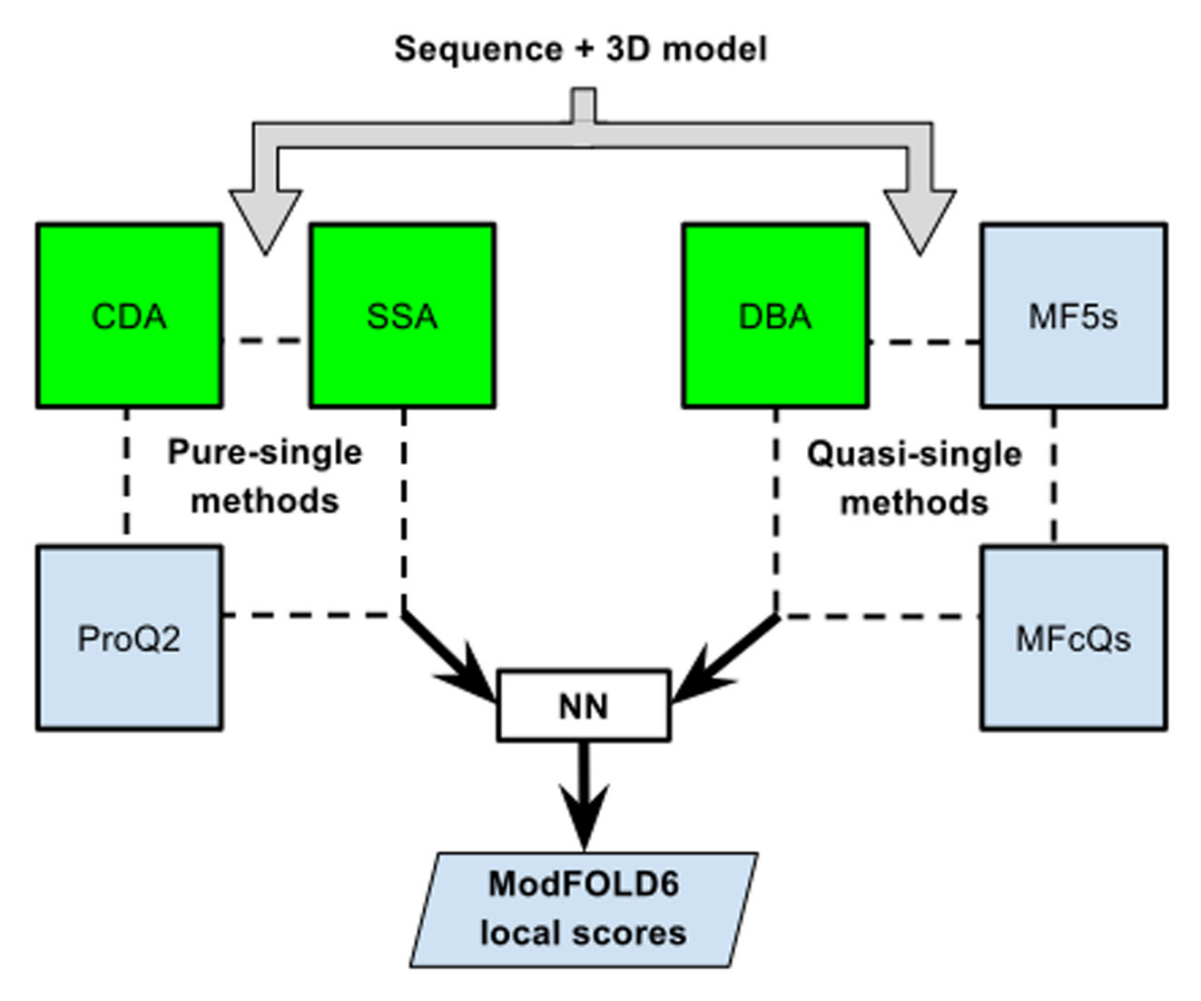
Flow of data for local quality assessment scoring in ModFOLD6. The target sequence and 3D model were evaluated with three pure-single model scoring methods (Secondary Structure Agreement (SSA), Contact Distance Agreement (CDA) and ProQ2) and three quasi-single model methods (Disorder B-factor Agreement (DBA), ModFOLD5_single (MF5s) and ModFOLDclustQ_single (MFcQs)). The new methods developed for ModFOLD6 are highlighted in green. The per-residue scores from all six methods were combined into a single residue score using an artificial neural network (see Supplementary Figure S1).

The ModFOLD6 server combines a pure-single and quasi-single model strategy for improved accuracy, which was originally developed for the CASP12 experiment. For ModFOLD version 6, our initial emphasis was on increasing the accuracy of per-residue assessments for single models. Each model was considered individually using three pure-single model methods, ProQ2 (8) and two newly developed methods: the Contact Distance Agreement (CDA) score and the Secondary Structure Agreement (SSA) score. Additionally, a set of 130 reference 3D models (generated using the latest version of IntFOLD (19–21)) was used to score models using three alternative quasi-single model methods: the Disorder B-factor Agreement (DBA) score, the ModFOLD5_single residue score and the ModFOLDclustQ_single residue score (Figure 1). An NN was then used to combine the component per-residue quality scores from each of the six alternative scoring methods, resulting in a final consensus of per-residue quality scores for each model.

Component per-residue/local quality scoring methods: (i) CDA is new pure-single model local QA method that relates to the agreement between the predicted residue contacts according to MetaPSICOV (24) and the model contacts, which are measured by the Euclidean distance (in Å) between residues in the 3D model. All pairs of residues in a model that were measured to be 8Å apart or less were considered to be in contact and the CDA score for each residue was calculated by the mean MetaPSICOV score for those model contacts. In other words, if residue *i* was measured to be in contact with both residue *j* and residue *k* in the model, and MetaPSICOV scores also existed for *ij* and *ik*, then the CDA score for residue *i* was taken as the mean MetaPSICOV score for *ij* and *ik*. Thus, *CDA = (∑p)/c*, where *p* is the MetaPSICOV score and *c* is simply the number of contacts for the residue in the model where a value for *p* also exists. (ii) SSA is a simple new pure-single model local QA method that relates to the agreement between the predicted secondary structure of each residue according to PSIPRED (25) and the secondary structure state of the residue in the model according to Dictionary of Secondary Structures of Proteins (DSSP) (26). Thus, *SSA = p_CHE_*, where, *p_CHE_* is simply the p-value from PSIPRED for the secondary structure state—coil (C), helix (H) or strand (E)—of the residue in the model according to DSSP. The eight DSSP states (H, I, G, E, B, S, T, -) were reduced to three states such that E (strand) and H (helix) were preserved and all other states were treated as C (coil). (iii) The local scores were also taken from the ProQ2 (8) method. (iv) The ModFOLD5_single local QA scores were calculated from the comparison of each model with the reference set of 130 models built by IntFOLD version 4, in a similar way to the ModFOLD4 (17) method acting in quasi-single model mode, with the predicted distances *d* converted back into residue similarities *S_r_*, thus: *S_r_ = 1/(1+(d/3.9)^2^)*. (v) The ModFOLDclustQ_single local QA scores were calculated in a similar way to ModFOLD5_single, however, in this case individual models were compared against the reference IntFOLD4 set using the local Q-score approach (16,27). (vi) DBA is a new quasi-single model QA method that relates to the agreement between the predicted disordered residues in the sequence according to DISOPRED3 (28) and the ModFOLD5_single predicted per-residue error. Thus, *DBA = 1-|S_r_-(1-P_d_)|*, where, *S_r_* is the ModFOLD5_single accuracy of the predicted residue for the model and *P_d_* is the probability of disorder according to DISOPRED3.

The final ModFOLD6 per-residue similarity scores were calculated using a simple multilayer NN (Supplementary Figure S1), which takes as its input a sliding window (size = 5) of per-residue scores from each of the 6 methods described above and outputs a single quality score for each residue in the model (30 inputs, 15 hidden, 1 output). The RSNNS package for R was used to construct the NN, which was trained using data derived from the evaluation of CASP11 server models. Similarity scores were converted back to distances in Ångströms, *d*, by rearranging the equation for S*r* above (Supplementary Figure S1).

Global scores were calculated by taking the mean per-residue scores (the sum of the per-residue similarity scores divided by the target sequence lengths) for each of the six individual component methods, described above and the NN consensus output (ModFOLD6). Furthermore, three additional quasi-single global model quality scores were generated for each model based on the original ModFOLDclust, ModFOLDclustQ and ModFOLDclust2 global scoring methods (16) (in a similar vein to the ModFOLD4_single and ModFOLD5_single *global* scores, tested in CASP10 (22) and CASP11 (23) respectively). Thus, we ended up with 10 alternative global QA scores, which could be combined in various ways in order to optimize for the different aspects of quality estimation (QE) (Supplementary Figure S2). The ModFOLD6 global score (the mean per-residue NN output score) considered alone was found to have a good balance of performance based on correlations of predicted and observed scores and rankings of the top models. The ModFOLD6_cor global score variant (calculated as: (ModFOLDclustQ_single_global + DBA_global + ModFOLD6_global)/3) was found to be an optimal combination for producing good correlations with the observed scores, i.e. the predicted global quality scores produced should produce closer to linear correlations with the observed global quality scores. The ModFOLD6_rank global score variant (calculated as: ModFOLDclustQ_single_global + ProQ2_global + CDA_global + DBA_global + SSA_global + ModFOLD6_global)/6) was found to be an optimal combination for ranking, i.e. the top ranked models (top 1) should be closer to the highest accuracy, but the relationship between predicted and observed scores may not be linear (Supplementary Figure S2).

## RESULTS AND DISCUSSION

### Server inputs and outputs

**Figure 2.**
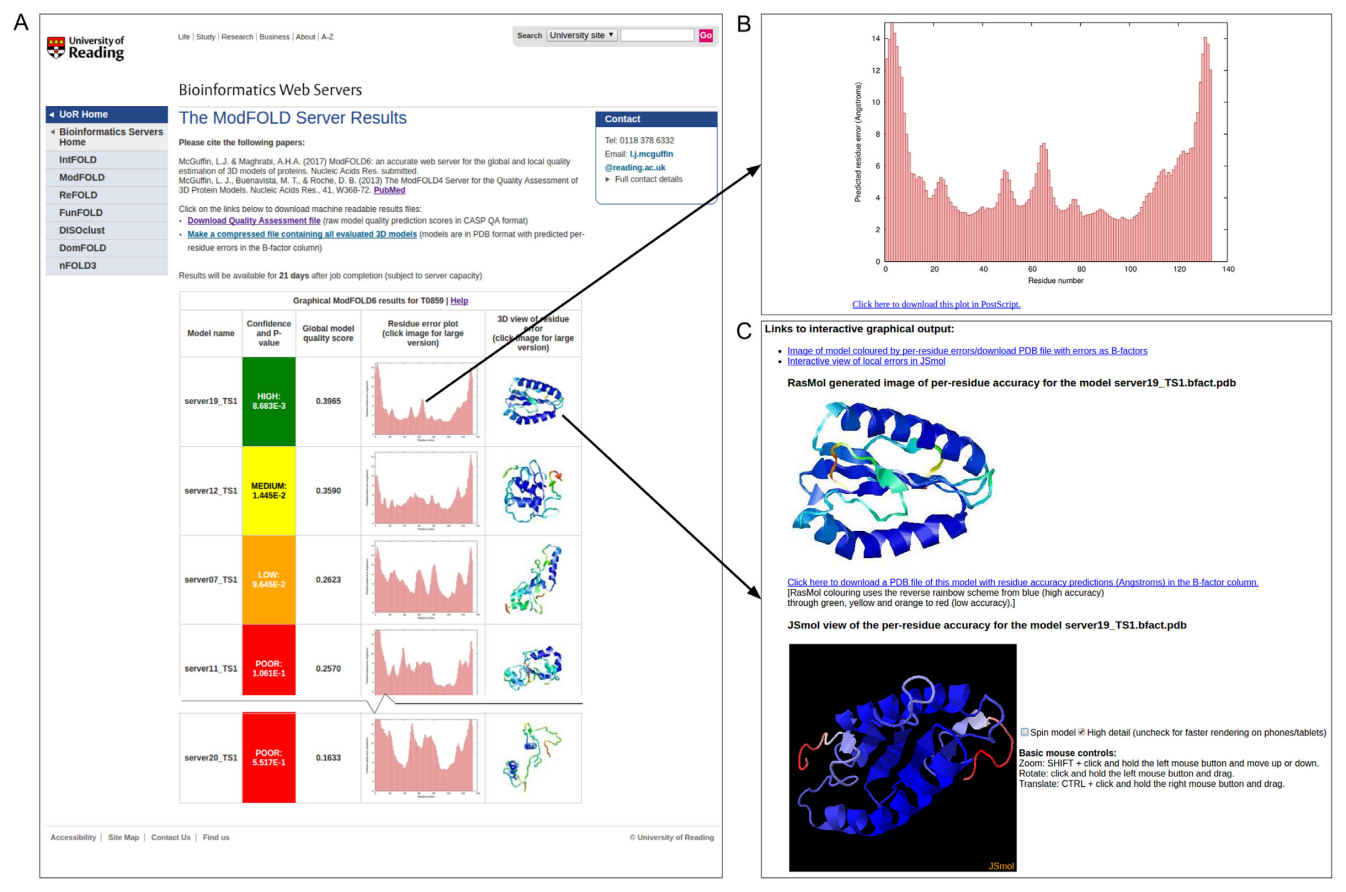
ModFOLD6 server results for models submitted to CASP12 generated for target T0859 (PDB ID: 5jzr). (**A**) An example of the graphical output from the server showing the main results page with a summary of the results from each method (truncated here to fit page). Clicking on the thumbnail images in the main table allows results to be visualized in more detail. (**B**) A histogram of the local or per-residue errors for the top ranked model, with the residue number on the x-axis and the predicted residue error (distance of the Cα atom from the native structure in Å) on the y-axis, which may be downloaded. (**C**) Interactive views of models, which can be manipulated in 3D using the JSmol/HTML5 framework and/or downloaded for local viewing.

The only required inputs to the ModFOLD6 server are the amino acid sequence for the target protein and a single 3D model (in PDB format) for evaluation. However, users may optionally upload multiple alternative models (as a compressed archive of PDB files), a name for their protein sequence and their email address. The server provides a clean and simple interface so that results can be easily interpreted by non-experts at a glance. The results page consists of a single table summarizing the quality assessment scores for each submitted model (Figure 2A). The prediction data in the table are represented graphically, with thumbnail images of the local error plots and annotated 3D models. Users can click through the images in the table in order to drill down into individual results and visualize annotated 3D models interactively in using the JSmol/HTML5 framework (Figure 2B and C). No plugins are required and, conveniently, interactive results may also be viewed on mobile devices.

Each row in the results table includes: a global score for the model, a *P*-value indicating the likelihood that the observed similarity between the model and native structure is random (TM-score < 0.2) and a plot of the local errors in the model (the predicted distance in Ångströms of each residue from the native structure) (Figure 2A). Conveniently, the server also inserts the predicted local quality scores into the B-factor column of the ATOM records for each submitted model and makes them available to download, either individually or as a compressed archive. The results table also includes a graphical view of each model coloured by predicted B-factors using the temperature scheme (Figure 2A and B). The raw machine readable data files for each set of predictions are also provided for developers, which comply with the CASP data standards.

### Independent benchmarking and cross validation

The ModFOLD6 server is continuously independently benchmarked for local QE performance using the CAMEO resource (29). At the time of writing, the CAMEO public QE data (http://www.cameo3d.org/) shows that ModFOLD6, and another unpublished method (QMEANDisCo), are currently the leading public QA methods for producing local (per-residue) quality scores, according to the lDDT measure over 6 months. Our common subset analysis using 6 months of CAMEO data prior to CASP12, verifies that the ModFOLD6 server is a significant improvement on our previous leading public ModFOLD4 method (17). Furthermore, results show that ModFOLD6 also outperforms the top publicly available published methods in terms of local quality (Table 1, Supplementary Figure S3 and Table S1).

**Table 1.** Independent benchmarking of local scoring with CAMEO using 6 months of common data comparing five publicly available published methods (177 025 common residues, 725 common models, 113 650 high quality residues, 63 375 low quality residues)

Method	AUC	StdErr	AUC 0–0.1	AUC 0–0.1 rescaled
ModFOLD6 (server18)	**0.8748**	0.00096	**0.0508**	**0.5081**
ModFOLD4 (server7)	0.8638	0.00099	0.0467	0.4669
ProQ2 (server 8)	0.8374	0.00107	0.0428	0.4283
Verify3d (server0)	0.7020	0.00134	0.0208	0.2081
Dfire v1.1 (server1)	0.6606	0.00138	0.0168	0.1675

Twenty-six weeks of data between 29 April 2016 and 21 October 2016 downloaded from http://www.cameo3d.org/. AUC = Area Under the ROC Curve. StdErr = Standard Error in AUC score. AUC 0-0.1 = Area Under the ROC curve with False Positive Rate ≤ 0.1. The table is sorted by the AUC score. See also Supplementary Tables S1–5 for independent local score benchmarks.

The ModFOLD6 server was also subjected to independent blind testing during the CASP12 experiment in 2016. We were invited to speak at the CASP12 meeting in Gaeta as one of the leading groups in the Estimation of Model Accuracy category. The ModFOLD6 server performed particularly well in terms of differentiating between good and bad models (Table 2), local scoring (Supplementary Tables S2–5) and assigning absolute global accuracy values (Supplementary Tables S6–9). The CASP12 data indicates that: ModFOLD6 ranks in top 10 in every benchmark of local score performance, it is the overall leading single model approach, it is competitive with the consensus/clustering approaches and it outperforms all pure-single model methods (Supplementary Tables S2–5). In terms of global scores, the ModFOLD6 variants were ranked within the top three for nearly every global benchmark using LDDT and CAD scores, as well as ranking within the top 10 according to other scores. (Table 2 and Supplementary Tables S6–10). The server was also a key factor contributing to our success in the Template Based Modelling category, where our group ranked in second position according to the assessors’ formula (http://www.predictioncenter.org/casp12/).

**Table 2.** Independent benchmarking of global scoring with official CASP12 data

			GDT_TS	LDDT	CAD(AA)	SG
Rank	Gr.Name	Gr.Model	AUC	AUC	AUC	AUC
1	**ModFOLD6_rank**	QA072_1	0.993	**0.99**	**0.926**	**0.962**
2	**ModFOLD6_cor**	QA360_1	**0.995**	0.988	0.885	0.949
3	**ModFOLD6**	QA201_1	0.994	0.988	0.878	0.944
4	qSVMQA	QA120_1	0.982	0.983	0.862	0.937
5	ProQ3	QA213_1	0.985	0.978	0.892	0.916
6	ProQ3_1_diso	QA095_1	0.982	0.978	0.891	0.922
7	ProQ3_1	QA302_1	0.981	0.977	0.889	0.917
8	ProQ2	QA203_1	0.944	0.971	0.921	0.932
9	MUfoldQA_S	QA334_1	0.977	0.968	0.898	0.913
10	MULTICOM-CLUSTER	QA287_1	0.956	0.968	0.893	0.921

The ability of methods to separate good models (accuracy score ≥ 50) from bad (<50) according to GDT_TS, LDDT, CAD and SG scores is evaluated using the Areas Under the Curve (AUC) (see http://predictioncenter.org/casp12/doc/presentations/CASP12_QA_AK.pdf). Only the top 10 methods are shown and the table is sorted using LDDT scores. The scores are calculated over all models for all targets (QA stage 1–select 20). The table is sorted by the LDDT AUC score. Data are from http://predictioncenter.org/casp12/qa_aucmcc.cgi. See also Supplementary Tables S5–10.

Prior to CASP12, the ModFOLD6 methods were also cross-validated using the CASP11 data to gauge performance versus the component methods, in terms of local (Supplementary Tables S11–13) and global scores (Supplementary Tables S14 and 15). In all target categories, the ModFOLD6 local scores significantly outperform the component methods. Similarly, significant performance gains can be made from combining component global scores, both in terms of cumulative GDT-TS of the top ranked models (with ModFOLD_rank) and in terms of assigning absolute accuracy values (with ModFOLD6_cor) (Supplementary Figure S2).

## CONCLUSION

The ModFOLD6 server provides users with intuitively presented, high accuracy estimates of local and global quality of 3D protein models. The ModFOLD6 server has been independently verified, via the CAMEO project, showing a significant improvement on our previous published server as well as taking the lead over other public published methods, in terms of local accuracy estimates. Furthermore, according to the recent CASP12 evaluation, the global scores produced by the ModFOLD6 sever methods rank among the best, outperforming other methods in terms of assigning absolute accuracy values to models and differentiating between good and bad models.

## Supplementary Material

Supplementary Data
