# Supplementary material for: ModFOLD6: an accurate web server for the global and local quality estimation of 3D protein models
Source: Nucleic Acids Res. 2017 Apr 29;45(Web Server issue):W416–21. doi: 10.1093/nar/gkx332 (PMC5570241; doi:10.1093/nar/gkx332)
Supplement: Supplementary Data [file gkx332_Supp.pdf]

Supplementary Figure S1. Details of neural network architecture and flow of data for local quality assessment scoring in ModFOLD6. Scores for each residue in the model are fed into the input layer, taken from the 6 local scoring methods using a sliding window of 5 residues (30 inputs). The hidden layer was made up of 15 hidden neurons and the network was trained to learn the output Si score of the residue in the model compared to the native structure according to the TM-score structural superposition ($S_i = 1/(1 + (d_i/d_0)^2)$, where S_i ranges from 0 to 1, di is the distance between structurally aligned residues and d0 is the distance threshold (3.9). 30 inputs and 15 hidden neurons was found to be an optimal architecture. No significant improvement was gained by further increasing the number of hidden neurons. The multilayer perceptron (MLP) function from RSNNS was used to build and train the network in R (https://cran.r-project.org/web/packages/RSNNS/).

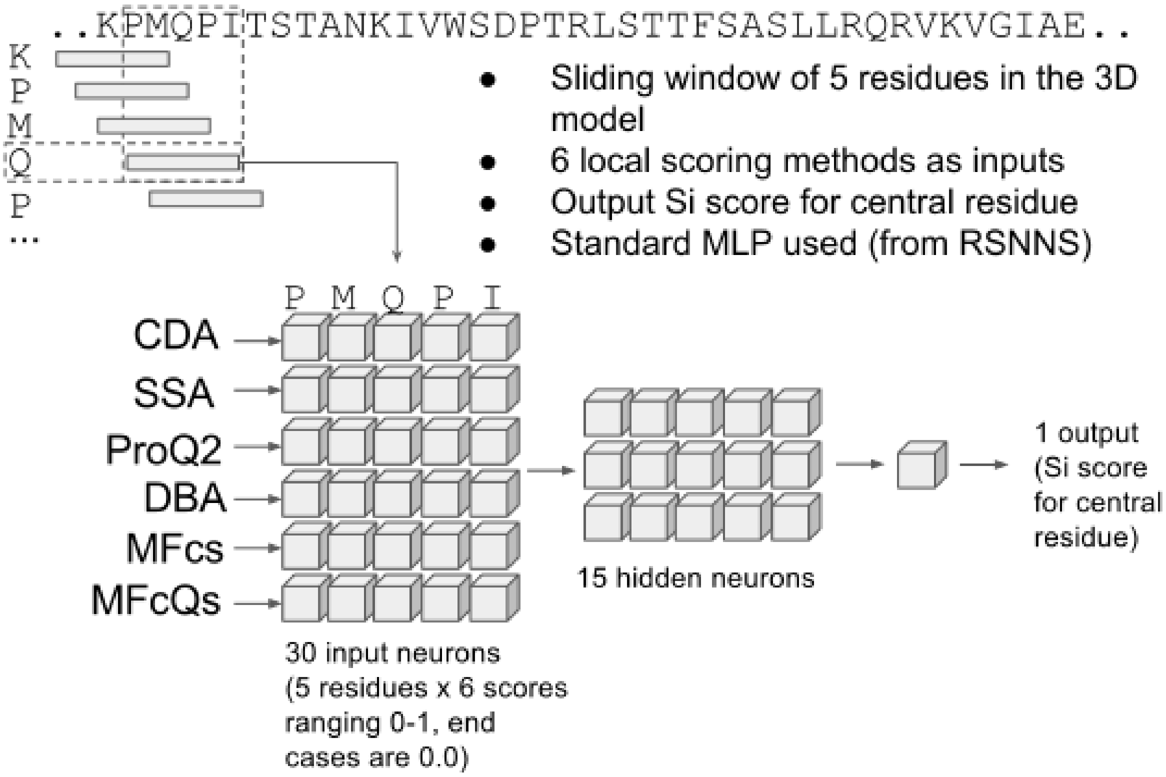

Supplementary Figure S2. Summary of global score benchmarks using CASP11 data. Visual comparison of ModFOLD6_rank, ModFOLD6 and ModFOLD6_cor - the three optimised global accuracy scores that may be selected by users on the ModFOLD6 server submission page.

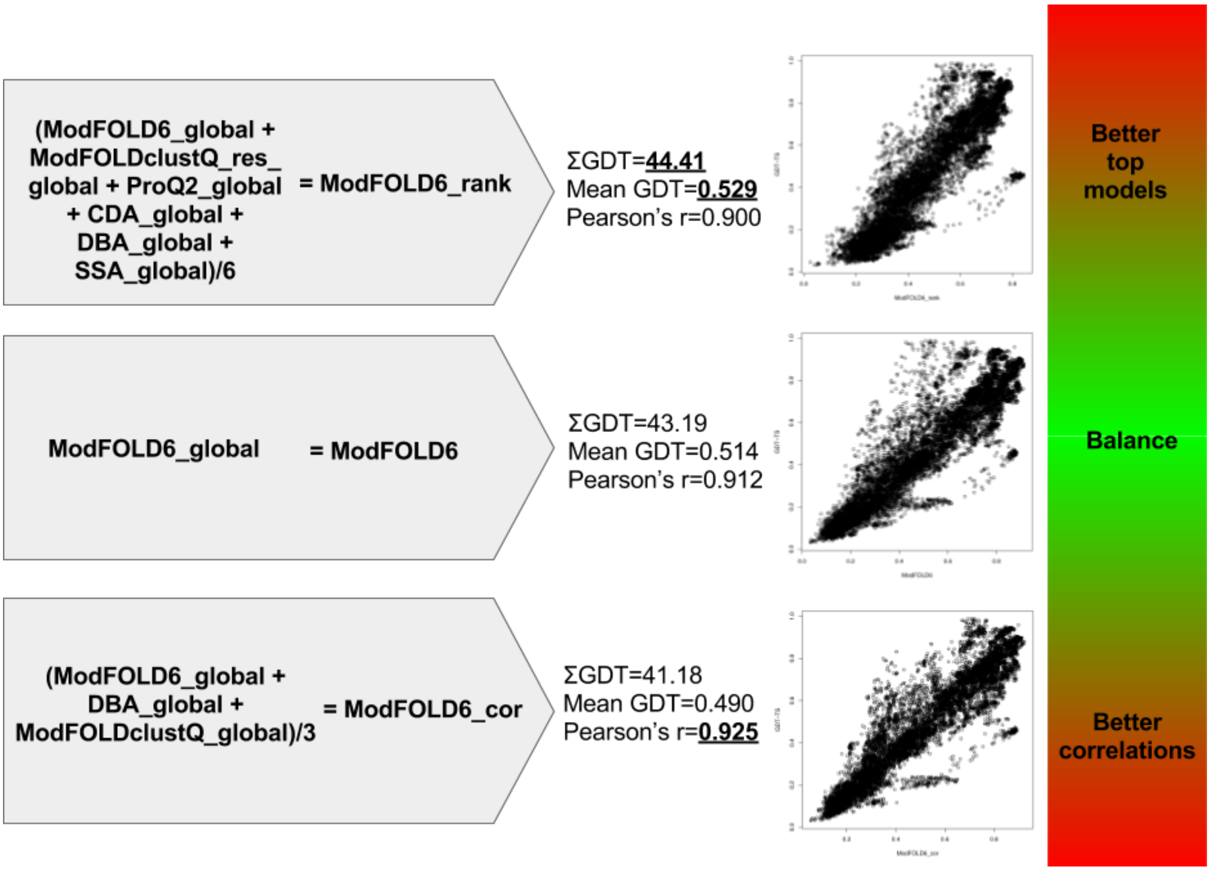

Supplementary Figure S3. Independent benchmarking of local scoring with CAMEO using 6 months of common data - ROC plots for data shown in Table 1 and Supplementary Table 2. A true positive is defined as a residue correctly identified to be low quality, with local LDDT <= 60. (**A**) Full ROC plot for common subset with 5 publicly available published methods. (**B**) ROC plot with FPR <= 0.1 for common subset including 5 publicly available published methods. (**C**) Full ROC plot for common subset including all 10 publicly available published methods. (**D**) ROC plot with FPR <= 0.1 for common subset including all 10 publicly available published methods.

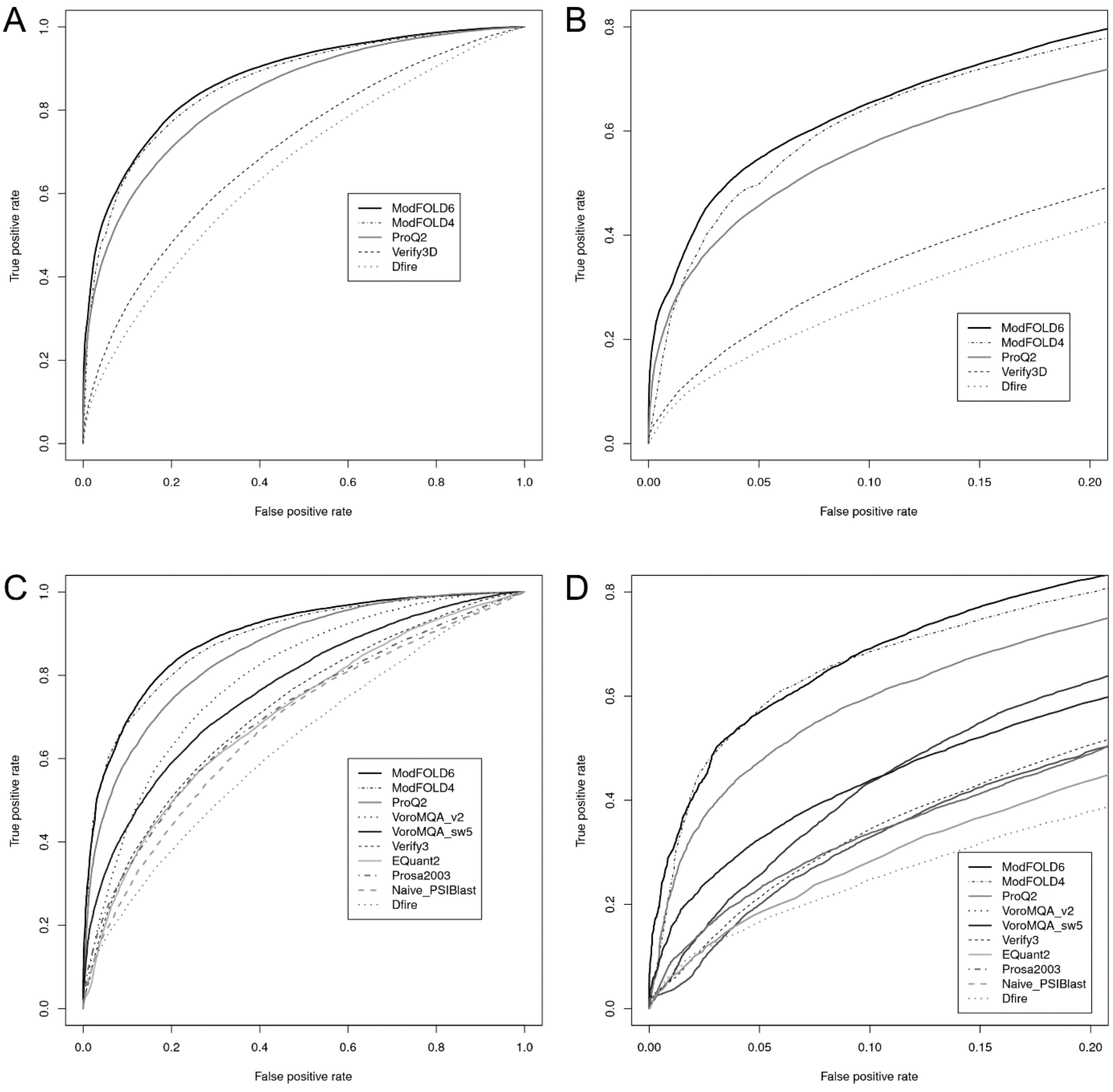

Supplementary Figure S4. Cross validation of ModFOLD6 local scores versus its component methods using CASP11 data - *ROC plots for the data shown in Supplementary Tables 11-13*. A true positive is defined as a residue correctly identified to be of low quality (> 3.5Å from the native structure). The full length chains were used for the official CASP11 QA analysis, and so they contain multiple domains of varying difficulty, with each domain being officially classified as either FM, TBM-hard or just TBM (easy). In order to demonstrate the performance of ModFOLD6 on easy, medium and hard CASP11 targets, we compare ROC plots for 3 different subsets of full length models: 1. Targets without any TBM-hard or FM domains (i.e. the models for easy targets), 2. Targets with TBM-hard domains (i.e. models for medium/hard targets) and 3. Targets with FM domains (i.e. models for hard targets). The analysis is carried out on all of the 84 targets with known structures. The targets with FM domains are: T0761, T0763, T0767, T0771, T0775, T0777, T0781, T0785, T0789, T0790, T0791, T0793, T0794, T0799, T0802, T0804, T0806, T0808, T0810, T0814, T0820, T0824, T0826, T0827, T0831, T0832, T0834, T0836, T0837, T0855. The targets with TBM-hard domains are: T0774, T0781, T0793, T0799, T0800, T0812, T0814, T0830, T0831, T0848. The targets without FM or TBM_hard domains include all remaining targets. Domain definitions are from: http://www.predictioncenter.org/casp11/domains_summary.cgi. The models from QA stage 1 and stage 2 were combined and all duplicate models (models from stage 1 occurring also in stage 2) were removed. (**A**) The full ROC plot for targets without FM or TBM-hard domains. (**B**) ROC plot with FPR <= 0.1 for targets without FM or TBM domains. (**C**) Full ROC plot for targets with TBM-hard domains. (**D**) ROC plot with FPR <= 0.1 for targets with TBM-hard domains. (**E**) Full ROC plot for targets with FM domains. (**F**) ROC plot with FPR <= 0.1 for targets with FM domains.

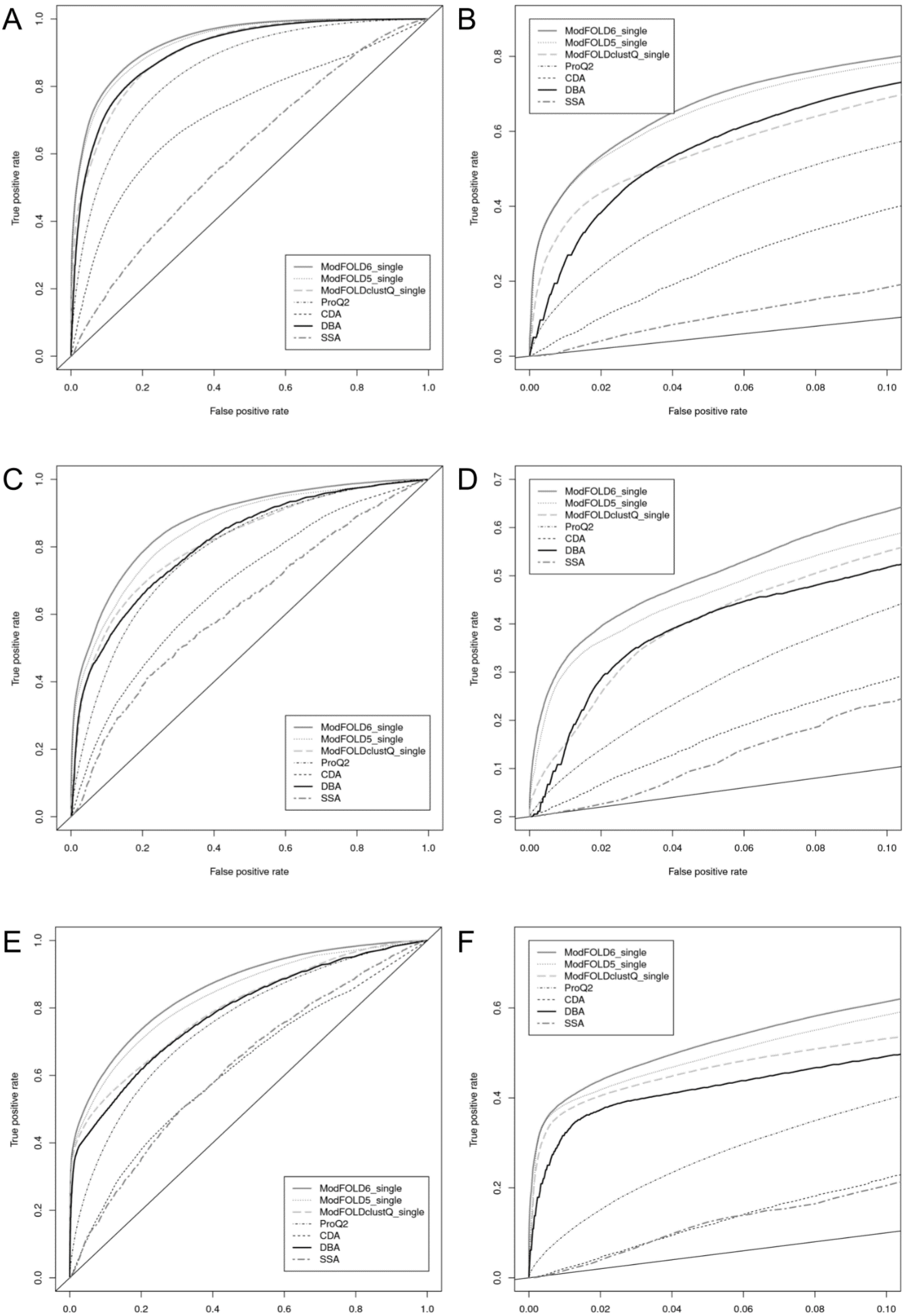
A
True positive rate
False positive rate
ModFOLD6_single
ModFOLD5_single
ModFOLDclustQ_single
ProQ2
CDA
DBA
SSA
B
True positive rate
False positive rate
ModFOLD6_single
ModFOLD5_single
ModFOLDclustQ_single
ProQ2
CDA
DBA
SSA
C
True positive rate
False positive rate
ModFOLD6_single
ModFOLD5_single
ModFOLDclustQ_single
ProQ2
CDA
DBA
SSA
D
True positive rate
False positive rate
ModFOLD6_single
ModFOLD5_single
ModFOLDclustQ_single
ProQ2
CDA
DBA
SSA
E
True positive rate
False positive rate
ModFOLD6_single
ModFOLD5_single
ModFOLDclustQ_single
ProQ2
CDA
DBA
SSA
F
True positive rate
False positive rate
ModFOLD6_single
ModFOLD5_single
ModFOLDclustQ_single
ProQ2
CDA
DBA
SSA

Supplementary Table S1. Independent benchmarking of local scoring with CAMEO using 6 months of common data including all 10 publicly available published methods (126 common models, 31076 common residues, 17984 high quality residues, 13092 low quality residues). 26 weeks of data between 2016-04-29 and 2016-10-21 downloaded from http://www.cameo3d.org/. AUC = Area Under ROC curve. StdErr = Standard Error in AUC score. AUC 0-0.1 = Area Under the ROC curve with False Positive Rate <= 0.1. Table is sorted by the AUC score.

Method	AUC	StdErr	AUC 0-0.1	AUC 0-0.1 rescaled
**ModFOLD6 (server18)**	**0.8921**	0.00200	**0.0525**	**0.5249**
ModFOLD4 (server7)	0.8830	0.00207	0.0519	0.5189
ProQ2 (server 8)	0.8552	0.00229	0.0437	0.4369
VoroMQA_v2 (server17)	0.7925	0.00267	0.0247	0.2472
VoroMQA_sw5 (server15)	0.7657	0.00280	0.0304	0.3036
Verify3d (server0)	0.7157	0.00300	0.0200	0.2003
EQuant 2 (server16)	0.7014	0.00305	0.0185	0.1848
Prosa2003 (server2)	0.7007	0.00305	0.0215	0.2148
Naive PSIBlast (server3)	0.6769	0.00312	0.0171	0.1712
Dfire v1.1 (server1)	0.6332	0.00322	0.0156	0.1564

Supplementary Table S2. Official CASP12 local QA evaluation (Corr/MCC, stage 1 - select 20). The top 10 groups are shown. Table is sorted by the Corr. score. Data are from http://predictioncenter.org/casp12/qa2_aucmcccorr.cgi.

**Rank**	**Gr.Name**	**Gr.Model**	**MCC(3.8)**	**MCC(5.0)**	**Corr.**
1	**ModFOLD6_rank**	QA072_1	**0.536**	**0.54**	**0.513**
2	**ModFOLD6**	QA201_1	**0.536**	**0.54**	**0.513**
3	**ModFOLD6_cor**	QA360_1	**0.536**	**0.54**	**0.513**
4	Pcons-net	QA432_1	0.457	0.511	0.381
5	Wallner	QA073_1	0.270	0.379	0.301
6	Pcons	QA089_1	0.220	0.307	0.275
7	ProQ3	QA213_1	0.332	0.313	0.216
8	ProQ3_1	QA302_1	0.342	0.340	0.204
9	ProQ3_1_diso	QA095_1	0.341	0.340	0.203
10	Wang1	QA132_1	0.188	0.164	0.198

Supplementary Table S3. Official CASP12 local QA evaluation (Corr/MCC, stage 2 - best 150). The top 10 groups are shown. Table is sorted by the Corr. score. Data are from http://predictioncenter.org/casp12/qa2_aucmcccorr.cgi.

**Rank**	**Gr.Name**	**Gr.Model**	**MCC(3.8)**	**MCC(5.0)**	**Corr.**	**AUC(3.8)**
1	Wallner	QA073_2	**0.756**	0.745	**0.697**	0.946
2	Pcons	QA089_2	0.755	0.76	0.688	0.945
3	Pcons-net	QA432_2	0.738	0.737	0.67	0.935
4	**ModFOLDclust2**	QA214_2	0.748	**0.785**	0.669	**0.949**

5	**ModFOLD6_cor**	QA360_2	0.73	0.719	0.657	0.938
6	**ModFOLD6_rank**	QA072_2	0.73	0.719	0.657	0.938
7	**ModFOLD6**	QA201_2	0.73	0.719	0.657	0.938
9	Pcomb-domain	QA411_2	0.717	0.744	0.642	0.939
8	Davis-EMAconsensus	QA034_2	0.717	0.773	0.635	0.948
10	ProQ3_1_diso	QA095_2	0.601	0.579	0.512	0.885

Supplementary Table S4. Official CASP12 local QA evaluation (Accuracy Self Estimates (ASE), stage 1 - select 20). The top 10 groups are shown. Table is sorted by the ASE score. Data are from http://predictioncenter.org/casp12/qa2_ase.cgi.

**Rank**	**Gr.Name**	**Gr.Model**	**ASE**
1	Davis-EMAconsensus	QA034_1	**85.831**
2	**ModFOLDclust2**	QA214_1	84.808
3	Pcons-net	QA432_1	84.805
4	Pcons	QA089_1	84.182
5	**ModFOLD6**	QA201_1	83.475
6	**ModFOLD6_cor**	QA360_1	83.473
7	**ModFOLD6_rank**	QA072_1	83.473
8	Pcomb-domain	QA411_1	83.146
9	Wallner	QA073_1	81.845
10	ZHOU-SPARKS-X	QA452_1	80.188

Supplementary Table S5. Official CASP12 local QA evaluation (ASE, stage 2 - best 150). The top 10 groups are shown. Table is sorted by the ASE score. Data are from http://predictioncenter.org/casp12/qa2_ase.cgi.

**Rank**	**Gr.Name**	**Gr.Model**	**ASE**
1	**ModFOLDclust2**	QA214_2	**87.032**
2	Davis-EMAconsensus	QA034_2	86.832
3	Pcons	QA089_2	86.679
4	Pcons-net	QA432_2	84.178
5	Wallner	QA073_2	84.17
6	**ModFOLD6**	QA201_2	83.852
7	**ModFOLD6_cor**	QA360_2	83.851
8	**ModFOLD6_rank**	QA072_2	83.851
9	Pcomb-domain	QA411_2	83.634
10	ProQ3_1_diso	QA095_2	79.158

Supplementary Table S6. Official CASP12 global QA evaluation (Differences in predicted versus observed scores, stage 1 - select 20). The top 10 groups are shown. Table is sorted by the LDDT score. Data are from http://predictioncenter.org/casp12/qa_diff_mqas.cgi.

Rank	Gr.Name	Gr.Model	GDT_TS	LDDT	CAD(AA)	SG
1	**ModFOLD6_cor**	QA360_1	6.697	**4.249**	17.461	9.931
2	**ModFOLD6_rank**	QA072_1	10.578	4.877	**13.368**	12.200
3	Pcomb-domain	QA411_1	8.56	4.987	17.031	10.413
4	QASproCL	QA267_1	9.107	5.432	16.59	11.624
5	**ModFOLD6**	QA201_1	**5.883**	5.813	19.241	**9.005**
6	MULTICOM-CLUSTER	QA287_1	10.222	5.835	14.943	12.013
7	Wang2	QA206_1	8.021	5.874	18.213	9.836
8	Deepfold-Contact	QA219_1	8.507	6.313	20.421	10.464
9	naive	QA109_1	8.507	6.313	20.421	10.464
10	DeepFold-Boom	QA223_1	8.507	6.313	20.421	10.464

Supplementary Table S7. Official CASP12 global QA evaluation (Differences in predicted versus observed scores, stage 2 - best 150). The top 10 groups are shown. Table is sorted by the LDDT score. Data are from http://predictioncenter.org/casp12/qa_diff_mqas.cgi.

Rank	Gr.Name	Gr.Model	GDT_TS	LDDT	CAD(AA)	SG
1	**ModFOLD6_rank**	QA072_2	9.754	**6.019**	**9.751**	12.732
2	**ModFOLD6_cor**	QA360_2	**6.748**	8.248	14.423	12.319
3	MULTICOM-CLUSTER	QA287_2	11.445	8.472	12.658	13.161
4	**ModFOLD6**	QA201_2	7.087	8.565	14.35	12.292
5	Pcomb-domain	QA411_2	9.839	8.842	11.312	13.275
6	qSVMQA	QA120_2	11.608	8.879	12.336	13.642
7	ProQ3_1	QA302_2	10.155	8.91	14.32	12.213
8	ProQ3_1_diso	QA095_2	10.159	8.931	14.778	**12.088**
9	MUfoldQA_S	QA334_2	8.898	9.053	16.343	12.268
10	ProQ3	QA213_2	11.418	9.14	15.006	12.622

Supplementary Table S8. Official CASP12 global QA evaluation (AUC/MCC, stage 1 - select 20). The ability of methods to separate good models (accuracy score >=50) from bad (<50) according to GDT_TS, LDDT, CAD and SG scores is evaluated using the Areas Under the Curve (AUC). The top 10 groups are shown Table is sorted by the LDDT AUC score. Data are from http://predictioncenter.org/casp12/qa_aucmcc.cgi.

			GDT_TS			LDDT			CAD(AA)			SG		
**Rank**	**Gr.Name**	**Gr.Model**	**MCC (40)**	**MCC (50)**	**AUC**	**MCC (40)**	**MCC (50)**	**AUC**	**MCC (40)**	**MCC (50)**	**AUC**	**MCC (40)**	**MCC (50)**	**AUC**
1	**ModFOLD6_rank**	QA072_1	0.613	0.814	0.993	**0.685**	**0.686**	**0.99**	0.382	0.502	**0.926**	**0.572**	0.523	**0.962**
2	**ModFOLD6_cor**	QA360_1	0.694	**0.863**	**0.995**	0.668	**0.686**	0.988	0.314	0.472	0.885	0.538	0.483	0.949
3	**ModFOLD6**	QA201_1	0.676	0.708	0.994	0.665	0.578	0.988	0.343	0.489	0.878	0.551	0.504	0.944

4	qSVMQA	QA120_1	0.521	0.579	0.982	0.587	0.562	0.983	0.399	**0.541**	0.862	0.544	0.525	0.937
5	ProQ3	QA213_1	0.579	0.625	0.985	0.611	0.53	0.978	0.314	0.524	0.892	0.491	**0.524**	0.916
6	ProQ3_1_diso	QA095_1	0.516	0.572	0.982	0.556	0.509	0.978	0.337	0.503	0.891	0.481	0.476	0.922
7	ProQ3_1	QA302_1	0.522	0.584	0.981	0.557	0.526	0.977	0.343	0.511	0.889	0.473	0.483	0.917
8	ProQ2	QA203_1	0.366	0.455	0.944	0.456	0.459	0.971	**0.48**	0.522	0.921	0.451	0.443	0.932
9	MUfoldQA_S	QA334_1	**0.716**	0.764	0.977	0.561	0.568	0.968	0.228	0.432	0.898	0.43	0.416	0.913
10	MULTICOM-CLUSTER	QA287_1	0.45	0.465	0.956	0.504	0.457	0.968	0.348	0.456	0.893	0.407	0.423	0.921

Supplementary Table S9. Official CASP12 global QA evaluation (AUC/MCC, stage 2 - best 150). The ability of methods to separate good models (accuracy score >=50) from bad (<50) according to GDT_TS, LDDT, CAD and SG scores is evaluated using the Areas Under the Curve (AUC). The top 10 groups are shown. Table is sorted by the LDDT AUC score. Data are from http://predictioncenter.org/casp12/qa_aucmcc.cgi.

			**GDT_TS**			**LDDT**			**CAD(AA)**			**SG**		
**Rank**	**Gr.Name**	**Gr.Model**	**MCC (40)**	**MCC (50)**	**AUC**	**MCC (40)**	**MCC (50)**	**AUC**	**MCC (40)**	**MCC (50)**	**AUC**	**MCC (40)**	**MCC (50)**	**AUC**
1	Wallner	QA073_2	0.721	0.734	**0.988**	0.707	0.745	**0.966**	0.351	0.592	0.923	0.665	0.665	**0.936**
2	Pcomb-domain	QA411_2	0.735	0.801	0.984	0.717	0.692	0.963	**0.531**	0.668	**0.925**	0.632	0.654	0.932
3	**ModFOLD6_rank**	QA072_2	0.763	0.843	0.983	0.74	**0.773**	0.962	0.486	**0.679**	**0.925**	0.639	0.675	0.929
4	QASproCL	QA267_2	0.788	0.783	0.987	0.733	0.676	0.958	0.493	0.635	0.906	0.629	0.635	0.928
5	MUfoldQA_C	QA318_2	0.812	0.844	0.982	0.746	0.727	0.958	0.442	0.65	0.902	0.652	0.654	0.927
6	Pcons	QA089_2	0.662	0.703	0.985	0.644	0.712	0.957	0.322	0.567	0.903	0.642	0.643	0.928
7	FDUBio	QA237_2	**0.835**	**0.872**	0.984	**0.773**	0.741	0.957	0.437	0.66	0.91	**0.673**	0.664	0.928
8	**ModFOLD6**	QA201_2	0.774	0.835	0.983	0.723	0.735	0.955	0.471	0.664	0.903	0.614	0.657	0.919
9	**ModFOLDclust2**	QA214_2	0.761	0.851	0.985	0.72	0.751	0.954	0.393	0.644	0.901	0.639	**0.677**	0.924
10	Pcons-net	QA432_2	0.66	0.66	0.979	0.633	0.662	0.954	0.324	0.537	0.923	0.612	0.616	0.925

Supplementary Table S10. Official CASP12 global QA evaluation (Difference from best - absolute differences stage 1 - select 20). For each score, only the targets with the best model scoring above the threshold (GDT_TS, SG: 40.0; LDDT, CAD(AA): 0.4) were considered. The top 10 groups shown. Table is sorted by the LDDT score. Data are from http://predictioncenter.org/casp12/qa_diff2best.cgi.

			**GDT_TS**		**LDDT**		**CAD(AA)**		**SG**	
**Rank**	**Gr.Name**	**Gr.Model**	**No. Targets**	**Score**	**No. Targets**	**Score**	**No. Targets**	**Score**	**No. Targets**	**Score**
1	MUfoldQA_C	QA318_1	47	**0.82**	45	**0.129**	66	**0.304**	43	**0**
2	**ModFOLD6_rank**	QA072_1	47	1.077	45	**0.129**	66	0.39	43	0.25
3	qSVMQA	QA120_1	47	1.186	45	**0.129**	65	0.487	43	0.325
4	**ModFOLD6_cor**	QA360_1	47	1.279	45	0.434	66	0.806	43	0.871
5	**ModFOLD6**	QA201_1	47	1.279	45	0.434	66	0.557	43	0.871
6	MUfoldQA_S	QA334_1	47	2.558	45	1.417	66	0.903	43	2.755
7	Pcons-net	QA432_1	42	2.949	41	1.434	57	0.767	40	1.973
8	QASproCL	QA267_1	47	3.644	45	1.619	66	1.502	43	1.938
9	SVMQA	QA208_1	47	3.557	45	1.723	65	0.739	43	2.93
10	ProQ3	QA213_1	47	4.244	45	2.148	66	1.113	43	3.103

Supplementary Table S11. Cross validation of ModFOLD6 versus its component methods using CASP11 data - local scores evaluated on stage1 and stage 2 models for targets with FM domains (861605 residues, 147428 high quality, 714177 low quality). A 3.5Å CA atom cut-off was used to define high quality residues (<=3.5Å are high quality, >3.5 Å low quality). Pearson = Pearson's r. Spearman - Spearman's rho. AUC = Area Under ROC Curve. AUC 0-0.1 = Area Under the ROC curve with False Positive Rate <= 0.1. StdErr = Standard Error in AUC score. Table is sorted by the AUC score. *See also Supplementary Figure S4.*

Method	Pearson	Spearman	AUC	AUC 0-0.1	StdErr
**ModFOLD6**	**0.6657**	**0.5478**	**0.8560**	**0.0505**	0.00064
ModFOLD5_single	0.6472	0.5285	0.8357	0.0479	0.00067
ModFOLDclustQ_single	0.6062	0.5225	0.7975	0.0450	0.00072
DBA	0.5543	0.3709	0.7856	0.0408	0.00074
ProQ2	0.3848	0.3642	0.7512	0.0250	0.00077
SSA	0.1571	0.1506	0.6242	0.0109	0.00084
CDA	0.1769	0.2012	0.6187	0.0114	0.00084

Supplementary Table S12. Cross validation of ModFOLD6 versus its component methods using CASP11 data - local scores evaluated on stage1 and stage 2 models for targets with TBM hard domains (344169 residues, 70689 high quality, 273480 low quality). A 3.5 Å cut-off was used to define high quality residues (<=3.5Å are high quality, >3.5 Å low quality). Pearson = Pearson's r. Spearman - Spearman's rho. AUC = Area Under ROC Curve. AUC 0-0.1 = Area Under the ROC curve with False Positive Rate <= 0.1. StdErr = Standard Error in AUC score. Table is sorted by the AUC score. *See also Supplementary Figure S4.*

Method	Pearson	Spearman	AUC	AUC 0-0.1	StdErr
**ModFOLD6**	**0.7111**	**0.6782**	**0.8736**	**0.0482**	0.00088
ModFOLD5_single	0.6664	0.6704	0.8512	0.0444	0.00094
ModFOLDclustQ_single	0.6212	0.6694	0.8150	0.0383	0.00102
DBA	0.5929	0.5839	0.8122	0.0374	0.00103
ProQ2	0.4653	0.4514	0.7888	0.0257	0.00107
CDA	0.2599	0.2808	0.6825	0.0154	0.00120
SSA	0.1697	0.1671	0.6226	0.0110	0.00123

Supplementary Table S13. Cross validation of ModFOLD6 versus its component methods using CASP11 data - local scores evaluated on stage1 and stage 2 models for targets without FM or TBM hard domains (2036888 residues, 1367703 high quality, 669185 low quality). A 3.5 Å cut-off was used to define high quality residues (<=3.5Å are high quality, >3.5 Å low quality). Pearson = Pearson's r. Spearman - Spearman's rho. AUC = Area Under ROC Curve. AUC 0-0.1 = Area Under the ROC curve with False Positive Rate <= 0.1. StdErr = Standard Error in AUC score. Table is sorted by the AUC score. *See also Supplementary Figure S4.*

Method	Pearson	Spearman	AUC	AUC 0-0.1	StdErr
**ModFOLD6**	**0.8337**	0.7975	**0.9340**	**0.0645**	0.00017

ModFOLD5_single	0.8192	**0.7982**	0.9282	0.0630	0.00017
DBA	0.7674	0.7411	0.9037	0.0524	0.00020
ModFOLDclustQ_single	0.7687	0.7494	0.9032	0.0526	0.00020
ProQ2	0.6467	0.6342	0.8496	0.0374	0.00026
CDA	0.3848	0.4024	0.7186	0.0220	0.00036
SSA	0.1835	0.1625	0.6040	0.0097	0.00041

Supplementary Table S14. Global score benchmarks using CASP11 data. ModFOLD6_rank versus component global scoring methods. Cumulative GDT scores and standard error. 84 targets with structures, models from QA round1 and round2 combined. The maximum possible GDT_TS is the cumulative score obtained by selecting the best model available for every target. The StdErr in GDT_TS is $\sigma/\sqrt{n}$, where σ is the standard deviation and n is the number of targets (84).Table is sorted by the ΣGDT_TS.

QA method used for model ranking	ΣGDT_TS	StdErr in GDT_TS
Maximum possible GDT_TS	48.4655	0.0273
**ModFOLD_rank**	**44.4149**	0.027**7**
ModFOLD6	43.1859	0.0280
ProQ2	42.9578	0.0270
ModFOLDclust2	42.6768	0.0294
CDA	40.4575	0.0281
ModFOLD5_single	40.0590	0.0291
DBA	40.0457	0.0290
ModFOLDclust2_single	40.0328	0.0292
ModFOLDclustQ_single	39.9194	0.0291
SSA	39.3166	0.0268
Random	37.8700	0.0284

Supplementary Table S15. Global score benchmarks using CASP11 data - correlations between predicted and observed global scores. ModFOLD6_cor versus component global scoring methods. R = Pearson's r. Rho - Spearman's rho. Tau = Kendall's tau. The analysis is carried out on all of the 84 targets with known structures. The models from QA stage 1 and stage 2 were combined and all duplicate models (models from stage 1 occurring also in stage 2) were removed. The table is sorted by the TMscore R values.

	GDT_HA			GDT			MaxSub			TMscore		
Method	R	Rho	Tau	R	Rho	Tau	R	Rho	Tau	R	Rho	Tau
ModFOLD6_cor	**0.9045**	**0.9288**	**0.7675**	**0.9250**	**0.9303**	**0.7793**	**0.9285**	**0.9335**	**0.7789**	**0.9266**	**0.9302**	**0.7816**
DBA_res_global	0.8962	0.9157	0.7405	0.9177	0.9192	0.7526	0.9230	0.9245	0.7585	0.9216	0.9212	0.7586

ModFOLD6_single_res_global	0.8793	0.9143	0.7451	0.9121	0.9181	0.7591	0.9133	0.9221	0.7608	0.9178	0.9196	0.7644
ModFOLDclust2_single_orig_global	0.8990	0.9234	0.7595	0.9152	0.9220	0.7649	0.9205	0.9256	0.7652	0.9157	0.9209	0.7647
ModFOLD5_single_orig_global	0.8860	0.9155	0.7469	0.9098	0.9155	0.7539	0.9160	0.9223	0.7618	0.9144	0.9162	0.7570
ModFOLD5_single_res_global	0.8905	0.9198	0.7540	0.9100	0.9203	0.7625	0.9186	0.9263	0.7682	0.9137	0.9211	0.7650
ModFOLDclustQ_single_res_global	0.8996	0.9200	0.7524	0.9054	0.9165	0.7536	0.9094	0.9161	0.7481	0.9003	0.9122	0.7490
ModFOLDclustQ_single_orig_global	0.8995	0.9200	0.7524	0.9053	0.9165	0.7536	0.9094	0.9161	0.7480	0.9003	0.9122	0.7490
ProQ2_res_global	0.6878	0.7319	0.5272	0.7182	0.7417	0.5404	0.7174	0.7427	0.5404	0.7239	0.7446	0.5452
CDA_res_global	0.6354	0.7192	0.5270	0.6703	0.7310	0.5407	0.6727	0.7300	0.5369	0.6746	0.7333	0.5431
SSA_res_global	0.5170	0.5595	0.3838	0.5348	0.5585	0.3840	0.5318	0.5601	0.3860	0.5324	0.5539	0.3816
